# New microbiological insights from the Bowland shale highlight heterogeneity of the hydraulically fractured shale microbiome

**DOI:** 10.1186/s40793-023-00465-1

**Published:** 2023-02-28

**Authors:** Natali Hernandez-Becerra, Lisa Cliffe, Wei Xiu, Christopher Boothman, Jonathan R. Lloyd, Sophie L. Nixon

**Affiliations:** 1 Williamson Research Centre for Molecular Environmental Science, Department of Earth and Environmental Sciences, The University of Manchester, Manchester, UK; 2 Manchester Institute of Biotechnology, The University of Manchester, Manchester, UK; 3 State Key Laboratory of Biogeology and Environmental Geology, China University of Geosciences (Beijing), Beijing, China

## Abstract

**Background:**

Hydraulically fractured shales offer a window into the deep biosphere, where hydraulic fracturing creates new microbial ecosystems kilometers beneath the surface of the Earth. Studying the microbial communities from flowback fluids that are assumed to inhabit these environments provides insights into their ecophysiology, and in particular their ability to survive in these extreme environments as well as their influence on site operation e.g. via problematic biofouling processes and/or biocorrosion. Over the past decade, research on fractured shale microbiology has focused on wells in North America, with a few additional reported studies conducted in China. To extend the knowledge in this area, we characterized the geochemistry and microbial ecology of two exploratory shale gas wells in the Bowland Shale, UK. We then employed a meta-analysis approach to compare geochemical and 16S rRNA gene sequencing data from our study site with previously published research from geographically distinct formations spanning China, Canada and the USA.

**Results:**

Our findings revealed that fluids recovered from exploratory wells in the Bowland are characterized by moderate salinity and high microbial diversity. The microbial community was dominated by lineages known to degrade hydrocarbons, including members of *Shewanellaceae*, *Marinobacteraceae, Halomonadaceae* and *Pseudomonadaceae*. Moreover, UK fractured shale communities lacked the usually dominant *Halanaerobium* lineages. From our meta-analysis, we infer that chloride concentrations play a dominant role in controlling microbial community composition. Spatio-temporal trends were also apparent, with different shale formations giving rise to communities of distinct diversity and composition.

**Conclusions:**

These findings highlight an unexpected level of compositional heterogeneity across fractured shale formations, which is not only relevant to inform management practices but also provides insight into the ability of diverse microbial consortia to tolerate the extreme conditions characteristic of the engineered deep subsurface.

**Supplementary Information:**

The online version contains supplementary material available at 10.1186/s40793-023-00465-1.

## Introduction

A significant fraction of the Earth’s microbial biomass is harbored in the deep subsurface [1], yet much of this remains unchartered. The subsurface is a challenging environment for life, thus microorganisms populating it must withstand extreme conditions including drought, high pressure, temperature, and salinity [2]. These microbial communities play important roles in biogeochemical cycling and there is bioprospective potential of novel biodiversity and their metabolic process.

The exploration of the deep subsurface in search of energy resources, such as shale gas, has offered the opportunity to probe the conditions that control microbial life in the deep subsurface. Shale gas is an economically relevant resource accessible through the application of technologies such as horizontal drilling and hydraulic fracturing. Shale reservoirs are horizontally drilled to maximize the area of contact with the rock and subsequently fractured by high pressure injection of water-based fluids to generate fissures in the shale. These fissures are held open by proppants such as sand, which allow natural gas to flow to the surface where it is collected. Input fluids typically contain a mixture of additives, many of which are organic and known to fuel microbial metabolism [3, 4]. Hydraulic fracturing thus introduces microorganisms and diverse energy sources, and creates new physico-geochemical conditions, that influence the composition of the microbial communities that inhabit the fractured shale environment [5].

Once hydraulic fracturing is completed, the injected fluids are recovered at the surface. The composition and volume of these fluids change gradually over time. Immediately after fracturing, flowback water consists mainly of injected fracking fluid mixed with salts and chemicals present in the formation [6]. As the well enters the production phase, the fluids recovered at the surface become more saline. Termed produced waters, these are comprised of a geogenic portion, consisting of the compounds native to the formation, and additives, included in injected fluids to stimulate formation of the fractures and aid production of gas [7]. As such, the chemical composition of both flowback and produced waters are highly variable and complex, containing high total dissolved solids (TDS), organics, heavy metals and naturally-occurring radioactive material (NORM) [8]. These fluids also carry microbiological signatures of the communities that inhabit fractured shales.

Prior studies of North American fractured shales have shown that temporal water chemistry variation is accompanied by microbial community structure shifts. For example, Mouser and colleagues (2016) summarized 16S rRNA gene sequencing data from input, flowback and produced fluids from five fractured shales in the US (Antrim, Barnett, Burkett, Marcellus and Haynesville) [5]. Their results revealed that, regardless of differences in operator procedures and shale deposition, strict and facultative anaerobic fermentative bacteria genera, such as *Marinobacter*, *Halomonas* and *Halanaerobium*, were widely distributed and dominant across hydraulically fractured shale formations. These findings were similar to numerous other microbiological studies conducted on fractured shales in North America [5, 7, 9–14].

Most of our knowledge about the microbiology of hydraulic fractured systems derives mainly from the study of North American shale plays. The exception is the work of Zhang et al*.* [15, 16] in the Sichuan Basin, China. Interestingly, these studies revealed a lack of organisms that were previously considered ubiquitous (and often dominant) in fractured shale environments, such as *Halanaerobium* species [5]. Instead, members of the genus *Shewanella* were found to dominate [15, 16]*.* Thus, characterizing unexplored fractured shales can aid identifying the processes underpinning the differential distribution of these industry-relevant taxa*.*

Understanding the microbiology and geochemistry of hydraulic fracturing flowback and produced fluids is essential for effective water management and microbial growth control and can thereby help achieve the safe and efficient extraction of shale gas. In so doing, we can gain fundamental insights on the microbial ecology of the fractured shale ecosystem. Our aim in this study is to expand on existing knowledge of hydraulic fractured systems by analyzing the geochemical and microbiological characteristics of flowback fluids from two wells in the Bowland shale, UK. These findings are placed into context with previously studied fractured shale communities using a meta-analysis approach, which serves to unveil how environmental factors may shape microbial communities across geographically distinct hydraulic fractured systems.

## Materials and methods

### Study site and sampling

The Bowland Shale is a Carboniferous formation located in the north of England, which sits on top of the Worston Shale Group, and below the Millstone Grit Group [17].This formation is subdivided into the Upper and Lower Bowland Shale, the former is a siliciclastic system while the latter is carbonate-dominated [18]. Flowback water samples were collected from two hydraulically fractured exploratory wells which targeted the Upper and Lower Bowland shale respectively [19], (hereinafter designated Bowland-1 and Bowland-2). Bowland-1 was drilled to a depth of ~ 2100 m, while Bowland-2 reached ~ 2300 m below ground [20]. Hydraulic fracturing occurred in intermittent stages to test the flow rates of natural gas (further details Additional file 1: Fig. S1). The fracturing fluid was comprised of water, silica sand, polyacrylamide (maximum concentration of 0.05%) and HCl (3%). UV disinfection treatment was applied to input fluids and flowback water when this was reused for further fracturing, but only for Bowland-1 [21]. The 7 samples from Bowland-1 were recovered from the well head upon flow back to the surface, data from this well has been reported elsewhere [22]. In contrast, due to restricted access, the 8 samples from Bowland-2 were taken from water storage tanks several weeks after flow back. Well operators collected the samples in sterile 500 ml Nalgene bottles which were filled to capacity to minimize headspace. Samples were stored at 4 °C on site until they were transported to the University of Manchester. In the laboratories, the samples were aliquoted for DNA sequencing and geochemical analyses. Aliquots of 100 ml were set aside for geochemical characterization (stored at 4 °C) and the rest of the sample was stored at -80 °C until molecular analyses were conducted.

### Chemical analysis

Anion concentrations were determined by ion chromatography (Dionex ICS5000 IC). Cation concentrations were measured by inductively coupled plasma mass spectrometry (Perkin Elmer ELAN 9000 ICP-MS). Prior to analysis, the samples were diluted 1:100 or 1:1000 due to high salinity levels. A subsample (10 ml) was filtered (0.45 μm) and analyzed for total organic carbon (TOC) with the combustion unit of a Shimazu TOC-V SSM-5000A. pH was measured with Denver Instrument digital meter and Fisherbrand FB68801 electrode.

### DNA extraction, 16S rRNA gene sequencing and data analysis

The biomass in the samples was concentrated by centrifugation at 4000 rpm (2670 g) in a BOECO C-28A centrifuge for 15 min. DNA from Bowland-1 flowback fluids was extracted from the concentrated samples with the DNeasy PowerSoil Pro Kit (Qiagen, Hilden, Germany). Bowland-2 concentrated samples were passed through a sterile 0.2 μm filter using vacuum filtration and the DNA was extracted from filters with a Dneasy PowerWater Kit (Qiagen, Hilden, Germany). Different DNA extraction protocols were followed for each of the wells due to low microbial loads. DNA extracts were quantified using Qubit (Life Technologies, Carlsbad, CA) following the protocols provided by the manufacturers. The 16S rRNA gene V4 region was amplified with the primer set 515F (5′-GTGYCAGCMGCCGCGGTAA-3′) and 806R (5′-GGACTACHVGGGTWTCTAAT-3′) [23]. Polymerase chain reaction (PCR) was set up as follows: initial denaturation step at 95 °C for 2 min, 36 cycles of melting (95 °C, 30 s), annealing (58 °C, 30 s), and extension (72 °C, 2 min), and final extension at 72 °C for 5 min. Amplified DNA was sequenced with the Illumina MiSeq platform [24].

Demultiplexed paired-end sequences were processed using QIIME2 version 2021.4 [25]. Denoising and amplicon sequence variant (ASVs) assignments were obtained with the *DADA2* plugin [26]. Taxonomy was assigned with the *q2-feature classifier* plugin [27] using the classify-sklearn naïve Bayes taxonomy classifier [28] against the Silva v138 99% reference sequence database [25, 29]. ASVs were inserted into the Silva reference tree using the SATé-enabled phylogenetic placement (SEPP) method [30] within the QIIME2 *fragment-insertion* plugin [31]. ASVs classified as mitochondria or chloroplast were flagged as potential artifacts and removed. Contaminant sequences identified in extraction, PCR, and sequencing controls were manually removed. R software version 4.1.1 (R Core Team, 2021) was used for statistical analyses and visualizations. The *microeco* package [32] was used to create an R6 class object which was used for downstream analyses. Sequences were rarefied (evenness = 28,100) to compute diversity metrics. Beta diversity distance matrix was calculated with the Bray–Curtis algorithm. Community composition differences were assessed using permutational multivariate analysis of the variance (PERMANOVA) with 999 permutations. The Mantel test was used to determine significant correlations (Pearson p < 0.05) between geochemical analytes and the Bray–Curtis distance matrix.

### Meta-analysis inclusion criteria, 16S rRNA gene sequencing data analysis

A meta-analysis was conducted to explore the broad trends of microbial community composition and geochemistry in the hydraulic fractured shale microbiome. Studies were included if they met the following criteria: (1) published before December 2021; (2) used high-throughput amplicon sequencing to target the V4 region of the16S rRNA genes in bacteria/archaea; and (3) provided corresponding geochemical characterization to the sequencing data. Several high-throughput studies were excluded since the sequencing protocols did not match the targeted single locus approach of our study (e.g., [11, 33]) or the raw data (sequences with quality information) were not publicly deposited or otherwise obtainable (e.g., [12, 34, 35]). All datasets included in the meta-analysis are detailed in (Additional file 2). Sequencing runs were downloaded using the function *fastq-dump* from the SRA Tool-box.

Sequencing datasets were processed separately following the same workflow to minimize technical bias, using QIIME2 and associated plugins as described above. The exception is a pre-processing step that trimmed off the primers with cutadapt [36]. When applying DADA2 for denoising and ASV generation, only forward reads were used with the same *trunc-len* parameter (120 bp) for all datasets. The representative sequences and ASV tables obtained from each dataset were then merged using the *feature-table merge* function. Taxonomic assignation and phylogenetic tree construction were based on these merged artifacts. The compiled dataset was imported to R and rarefied (evenness = 1259). Alpha and beta diversity metrics were calculated with the *microeco* package [32]. Missing values in the geochemical dataset were imputed using the *char* method from the mice package [37]. Redundancy analysis (RDA) was used to determine which geochemical variables influence changes in microbial community composition using the *vegan* package. Network analysis was used to visualize microbial interactions across formations. This analysis was based on a pruned dataset that contained only ASVs comprising more than 1% of the sequences recovered from at least one sample. The SPIEC-EASI statistical method was used to construct the network, which combines a transformation for compositional correction of the ASVs and graphical model to infer the network based on the inverse covariance matrix [38]. The method was implemented with the *trans-network* class from the *microeco* package. Network attributes were obtained with the same package. Nodes classified based on within-module connectivity and among-module connectivity using the thresholds described elsewhere [39], were obtained with the same package. The network was visualized using Gephi version 0.9.4.

## Results and discussion

### Bowland shale flowback fluids are moderately saline and dominated by *Marinobacter *and *Shewanella*

Flowback fluids from the first exploratory shale gas wells in the UK were analyzed to gain insight into the microbial ecology and geochemistry of the fractured Bowland shale. Well differences, including frac stages and microbial control, provided the opportunity to explore the influence of operational procedures in the shale microbiome.

**Fig. 1:**
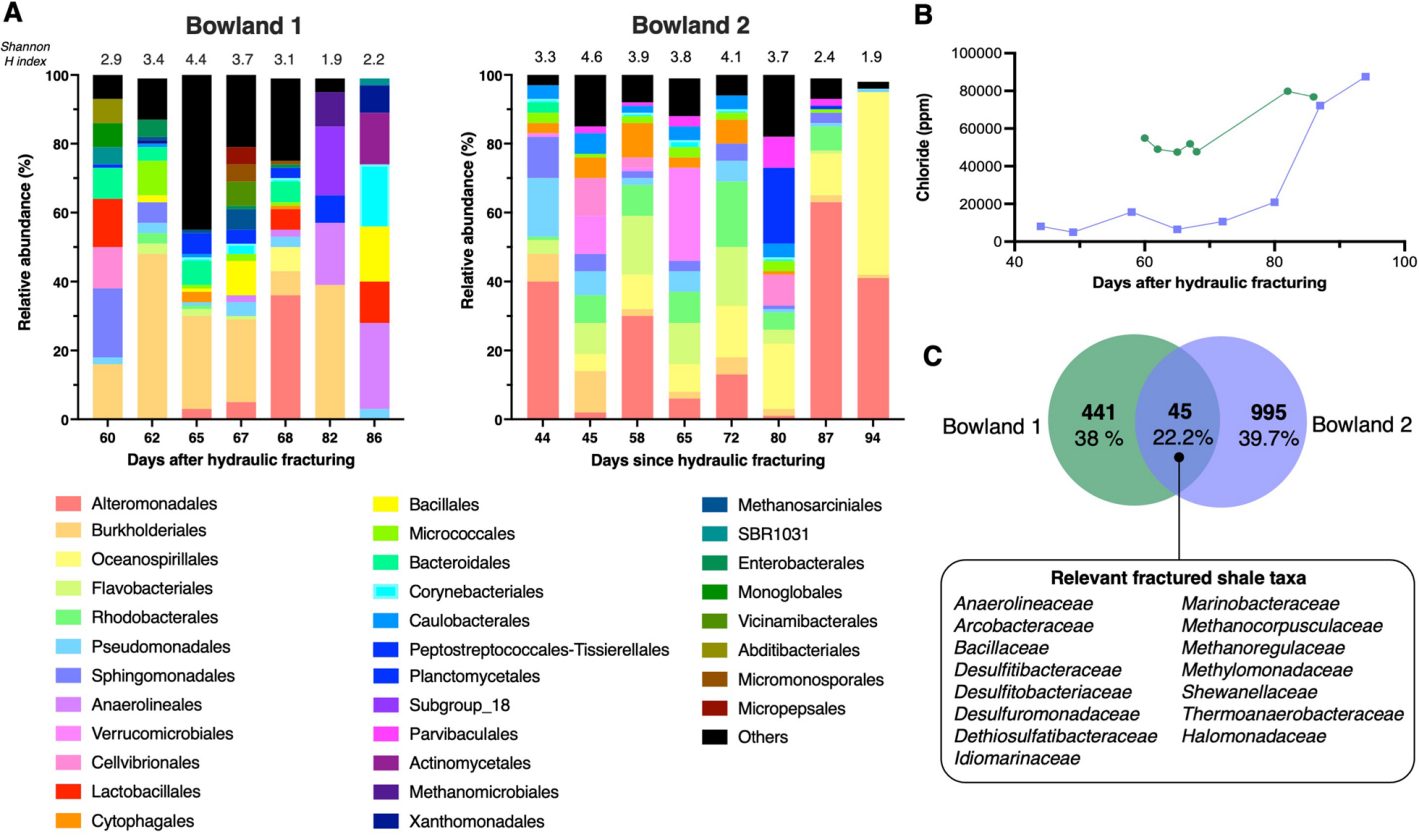
Microbial community composition of flowback fluids derived from the Bowland shale exploration wells. **A** Relative abundance of microbial classes in flowback fluids from the Bowland shale. All classes that represent ≥ 5% of sequences from any sample are listed on the bar plot, the rest are grouped as “Other”. **B** Temporal changes in chloride (ppm) concentration in the Bowland-1 (green) and Bowland-2 (purple) flowback fluids. **C** Venn diagram depicting the number of shared and unique ASVs (percentage of the total number of sequences) in each well. Relevant shared taxa at family level, i.e., organisms associated with shale systems and hydrocarbon reservoirs reported in previous studies, are indicated in the box

The geochemical characterization of the flowback water revealed that the pH remained circumneutral, varying from 6 to 7.5 (Additional file 1: Fig. S2). In general, flowback fluids from Bowland-1 exhibited higher concentrations of ionic species compared to Bowland-2. The exception was calcium which was three times higher in Bowland-2 (Additional file 1: Fig. S2). This can be expected from the carbonate-dominated Lower Bowland shale [40], which was the targeted formation for this well. The most abundant ion, chloride, ranged from 54, 883 mg/L in the earliest fluid sample (day 60) to 76,776 mg/L in the latest sample (day 86) from Bowland-1. While, in Bowland-2 the earliest flowback (day 44) recorded a chloride concentration of 8,109 mg/L and 87, 499 mg/L the latest flowback (day 94) (Fig. 1B). Overall, Bowland-1 had a significantly higher average chloride of 58 204 mg/L compared to 28 295 mg/L in Bowland-2 (Additional file 1: Fig. S3).

DNA yields contrasted strongly between the two wells, with higher yields achieved from Bowland-2 compared with Bowland-1 (Additional file 1: Fig. S3). We note that Bowland-1 input fluids were UV-sterilized and this is likely to explain the difference in DNA yields. In addition, Bowland-2 samples were collected from storage tanks several weeks after they were recovered at the well pad, and hence are likely to reflect a degree of microbial growth during storage.

Microbial community composition was assessed with 16S rRNA gene amplicon sequencing. A total of 1440 ASVs were identified, of which only 45 (representing 22% of total sequences) were shared between the two wells (Fig. 1C). Taxonomic profiling showed a unique community structure in each well, with 25 dominant orders identified in Bowland-1 and 12 in Bowland-2. Sequences from Bowland-1 flowback fluids were predominantly assigned to the orders *Burkholderiales* (7–48%), *Anaerolineales* (2–25%) and *Alteromonadales* (3–36%). Within the *Burkholderiales*, sequences affiliated with the families *Comamonadaceae* (2–22%), and *Burkholderiaceae* (1–41%) comprised a large fraction of the community (Fig. 1A). Sequences assigned to the *Anaerolinea* (25%) genus were prominent within the *Anaerolineales,* and *Shewanellaceae* (2–36%) was the was the dominant family from the *Alteromonadales*. The majority of Bowland-2 sequences were assigned to the orders *Alteromonadales* (2–63%), *Oceanospirillales* (5–53%) and *Flavobacteriales* (4–17%) (Fig. 1A). The sequences affiliated with the families *Marinobacteraceae* (7–55%), and *Shewanellaceae* (1–26%) dominated within the *Alteromonadales*. Within the *Oceanospirillales*, *Halomonadaceae* was prominent (5–49%). Sequences assigned to the genus *Flavobacterium* (1–13%) were dominant within the *Flavobacteriales*. Sequences assigned to the Archean orders *Methanomicrobiales* and *Methanosarciniales* were detected in fluids from Bowland-1 (ranging from 1 to 10%), while they were only detected in very low abundances in Bowland-2 fluids (0.01–0.09%).

NMDS ordination analysis of sequencing results from the Bowland shale gas wells identified clustering by well, and analysis using PERMANOVA confirmed significative differences between the wells (Additional file 1: Fig. S4). Microbial communities of the Bowland-2 flowback fluids, sampled from storage tanks, exhibited significantly greater ASV richness than the communities recovered from Bowland-1, which were collected from the separator (Additional file 1: Fig. S2). These observations concur with the pattern detected in the Sichuan formation, where compositional differences were detected between separators and storage tanks [16]. In particular, the relative abundance of sulfate-reducing bacteria increased from separators (0.39%) to storage tanks (2.37%) [16]. Besides the sampling point, operational practices might also account for the differences observed in microbial community composition. For instance, well operators reported that UV disinfection treatment was successfully applied to the recycled input fluids used in Bowland-1, while the technology was not used in Bowland-2 fluids. The differences in DNA yields recovered from the two wells confirm that Bowland-1 fluids had a lower microbial biomass (Additional file 1: Fig. S2). We therefore infer that UV sterilisation applied to Bowland-1 was successful in controlling microbial loads, evidenced from the below detection yields of DNA recovered from this well.

Pearson correlation tests revealed a significant positive correlation between the Bray–Curtis distance matrix and pH, chloride, bromide, and strontium (Additional file 1: Fig. S3). Similar to our analysis, prior studies have reported shifts in fractured shale microbial community composition associated with variation in the water geochemistry, with TDS, TOC and sulfate considered some of the key parameters driving microbial diversity in previously studied fractured shale microbial communities [7, 9, 41, 42].

Microbial communities in hydraulically fractured systems typically exhibit a marked temporal pattern, shifting from a diverse assemblage of predominantly aerobic microorganisms resembling input fluids in the early flowback points to low diversity, facultative or anaerobic halotolerant communities inhabiting the highly saline produced waters [5, 7, 9–11, 14]. Unlike most hydraulically fractured sites previously studied, the Bowland exploratory wells were fractured in intermittent stages, which prolonged the recovery of flowback fluids for over three months, and they did not reach the production phase. During this long flowback period we identified changes in the microbial community composition. Communities in the earlier flowback fluids were more diverse compared with the later time points, with Shannon diversity decreasing from 2.9 to 2.2 and 3.3 to 1.9 in Bowland-1 and Bowland-2, respectively (Fig. 1A). This decrease in diversity coincided with an increase in chloride concentration peaking at 87,499 mg/L for the day 82 sample from Bowland-1 and 15,697 mg/L in the latest sample (day 94) from Bowland-2 (Fig. 1B). The taxonomic assignment of the ASVs highlighted distinct groups of microorganisms dominating the later flowbacks. In Bowland-1 (day 82 and 86), the sequences were most closely affiliated with the family *Gallionellaceae*, dominated by genera Subgroup 18, *Leviliinea*, *Anaerolinea* and *Anaerobacillus*. In Bowland-2, flowback fluids from day 87 and 94 were dominated by *Halomonas* and *Marinobact*er species. Interestingly, even in our later flowback samples characterized by high chloride concentrations comparable to produced waters from other formations (e.g., [43]), members of the usually dominant *Halanaerobium* genus were not detected.

Many hydraulic fracturing additives are organic, and estimates suggest more than one-third of these organic chemicals can be degraded [44]. For instance, urea is commonly used as a friction reducer and may be consumed as a carbon and nitrogen source by members of the *Marinobacter* and *Arcobacter* genera [45]. Glycine betaine is used as a surfactant and is known to serves as a key metabolite for salinity adaptation [42] but also it can be fermented by *Halanaerobium* species [46]. Further, the breakdown products from this metabolism have been shown to subsequently fuel methylotrophic methane production by *Methanohalophilus* [47]*.* Polyethene glycols, which are added as surfactants, emulsifiers and crosslinkers, are known to be biodegraded both aerobically and anaerobically [48]. Regardless of additives used in the hydraulic fracturing of shale gas wells, drilling muds used to drill these wells are also rich in additives, including weighting agents and thickeners that have been shown to stimulate microbial growth [49]. Together, these observations indicate that additives may play an important role in supporting microbial communities in hydraulically fractured shale formations.

In contrast to the additive-rich fracturing fluids commonly used in shale gas extraction, the input fluids injected into the Bowland shale during hydraulic fracturing were comparatively lean, comprising only water, silica sand, polyacrylamide (maximum concentration of 0.05%) and HCl (3%) (Additional file 1: Fig. S1). In the absence of other more bioavailable additives, we hypothesize that this lean composition may lead to a community supported by hydrocarbon metabolism, utilizing hydrocarbons from the formation itself, instead of being heavily influenced by additive chemistry in input fluids. The taxonomic analysis identified several lineages that have previously been implicated with hydrocarbon degradation, such as *Alcanivorax*, *Marinobacter*, *Halomonas*, *Thalassolituus*, *Flavobacterium* and *Idiomarina* [50–52]. Significantly, isolates of the genus *Marinobacter* recovered from the Utica Point Pleasant formation have the potential to use aliphatic and aromatic hydrocarbons [45].

### Salinity is a key driver of fractured shale microbiome composition

**Fig. 2:**
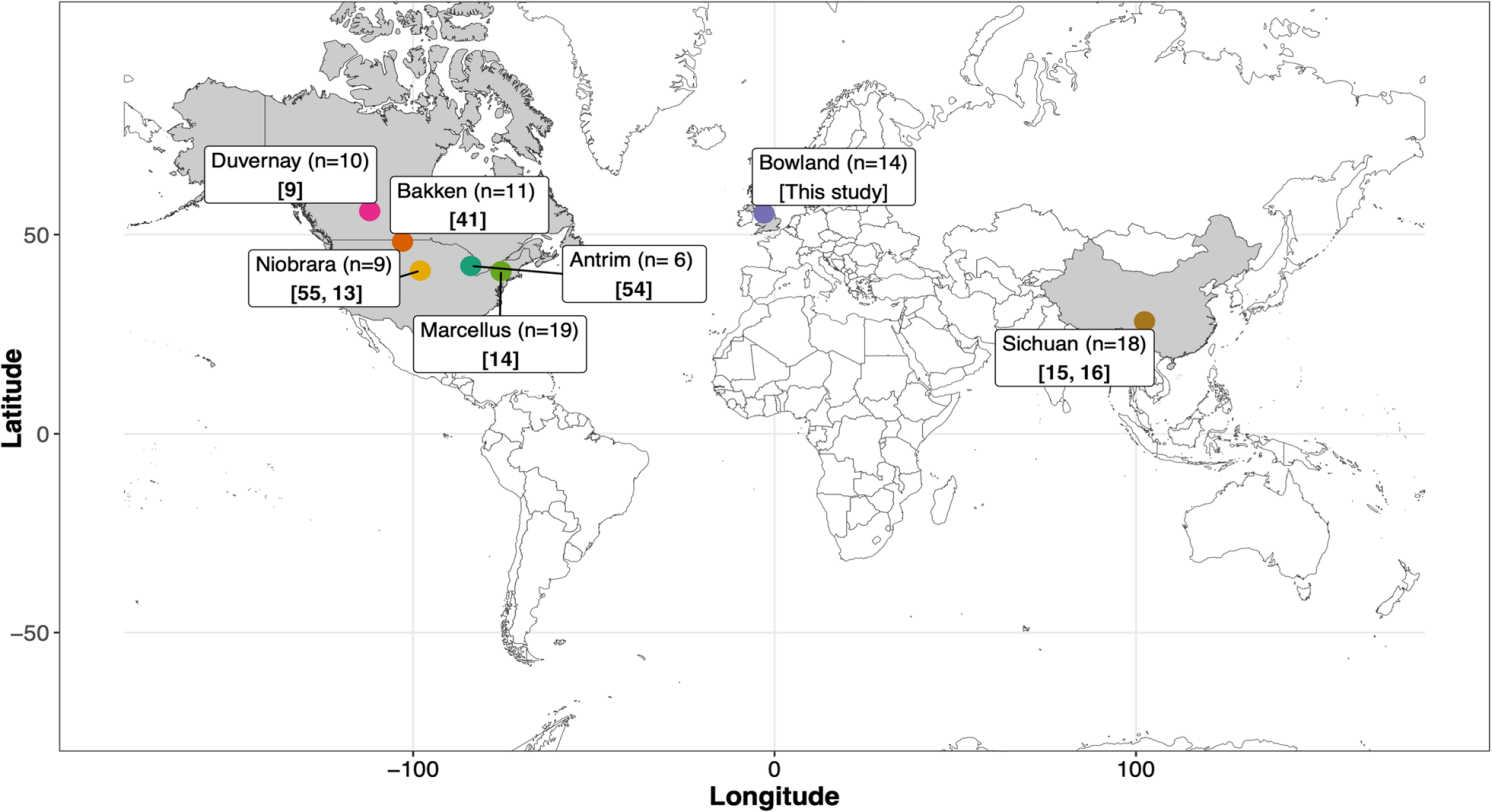
Map of the location of the datasets examined in the meta-analysis. Number of samples per formation are indicated in parentheses and corresponding references in square brackets. [54] Stemple et al., 2021; [41] Tinker et al., 2020; [9 Zhong et al., 2019; [14] Cluff et al., 2014; [55, 13] Rosenblum et al. 2017, Harris et al., 2018; [15, 16] Zhang et al., 2017, 2020

Understanding the dynamics of the fractured shale microbiome sheds light on microbial survival in the deep subsurface and is key to improving operational practices of these engineered systems. Generally, microbial activity in the engineered subsurface is associated with detrimental processes including bioclogging, sulfide production and microbial induced corrosion [12, 53]. Our meta-analysis builds on previous work to analyze spatio-temporal distributions of microorganisms across geographically distinct formations [9, 13–16, 41, 54, 55]. The dataset compiled here spans seven fractured shale formations (Fig. 2), and was comprised of 87 flowback or produced water samples, ranging from 0 to 10,950 days after hydraulic fracturing, with paired 16S rRNA gene V4 amplicon sequencing and geochemical data.

**Fig. 3:**
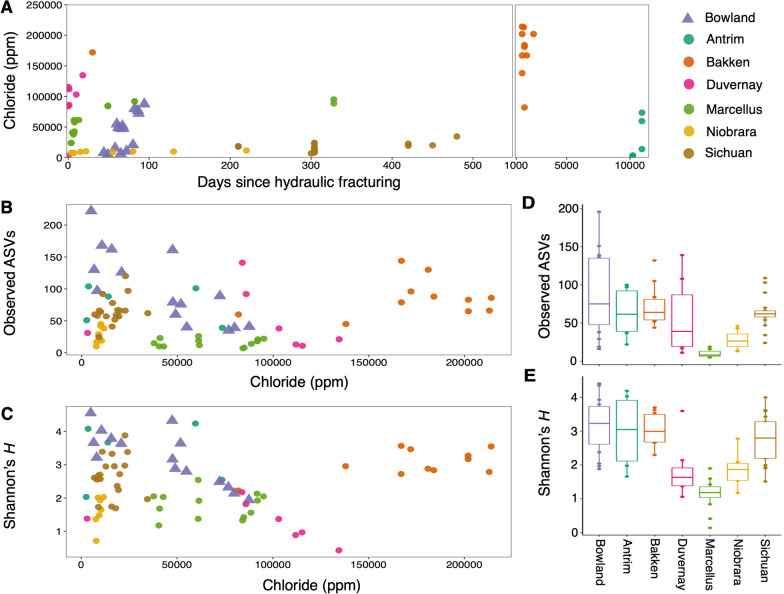
Temporal and geographical patterns across shale formations. **A** Chloride concentration in flowback and produced fluids across shales. **B** Observed ASVs and **C** Shannon values variation in function of chloride concentration. **D** Boxplot comparison of observed ASVs and **E** Shannon (H′)

Despite geographical and depositional differences, hydraulically fractured shales share a temporal variation of TDS concentration increasing as the wells transition from the flowback to the production phase. Previous studies have noted that the dominant ions (Cl, Na, K and Ca) increase dramatically over time in the fluids [7, 11, 14, 55, 56]. This is due to the liberation of brines in the formations and the dissolution of salts present in the rock matrix upon injection of freshwater-based fluids [8, 43, 57]. Consistent with previous studies, the flowback and produced water derived from geographically distinct shales included in our meta-analysis exhibit similar temporal shifts. Generally, the concentration of chloride (the principal component of TDS) increased over time (Fig. 3A). Moreover, we identified a broad range of salinity levels across formations. Water is often classified in discrete categories according to its salinity [58–60]. Here we used the following categories based on NaCl concentration: saline (2000–10,000 mg/L), highly saline (10,000–60,000 mg/L) and brine (over 60,000 mg/L). Fluids recovered from the Niobrara and Sichuan formations exhibited lower chloride concentrations with an average of 15,264 mg/L and 25,901 mg/L respectively. In contrast, the Antrim and Bowland shale fluids can be considered highly saline with chloride concentrations averaging 40,536 mg/L and 53,432 mg/L, respectively. Brine levels were consistently reached by the fluids from the Marcellus, Duvernay and Bakken formations (averaging 77,290 mg/L; 121,025 mg/L and 257,100 mg/L; respectively).

TDS is one of the main drivers of microbial dynamics in the fractured shale microbiome, since the increase in salinity overtime constrains habitability of the system to halotolerant organisms [9, 14, 55]. We looked for evidence of this in our meta-study. Among the normalized datasets, the number of observed ASVs varied between 7 and 222 and a negative trend was identified between ASVs richness and chloride concentration (Fig. 3B), as expected. Similarly, microbial community evenness assessed with Shannon’s *H* indices decreased in samples with higher concentrations of chloride and ranged from 0.42 to 4.5 (Fig. 3C). Significantly, the Bakken shale exhibited high microbial richness despite exhibiting highest salinity across the compiled dataset (Fig. 3). The advanced age of the Bakken shale wells analyzed has previously been pointed to as a factor influencing the stability of these communities [41]. Besides the temporal variability, geographically distinct formations exhibit particular microbial traits. The communities associated with the fluids derived from the Antrim, Bakken, Bowland, Duvernay and Sichuan formations presented a significantly higher ASV richness than the communities associated with the Niobrara and Marcellus shales (Fig. 3D). Formations characterized with lower richness were significantly more uneven with average *H′* values ranging from 1.6 to 1.8 (Fig. 3E).

The taxonomic analysis of sequencing data revealed the presence of 15 dominant bacterial phyla and 2 archaeal phyla across the datasets. *Proteobacteria, Firmicutes, Halanaerobiaeota* and *Desulfobacterota* were the main bacterial phyla identified. Members of *Euryarchaeota* and *Halobacterota* represented the two dominant archaeal phyla (Additional file 1: Fig. S6).

**Fig. 4:**
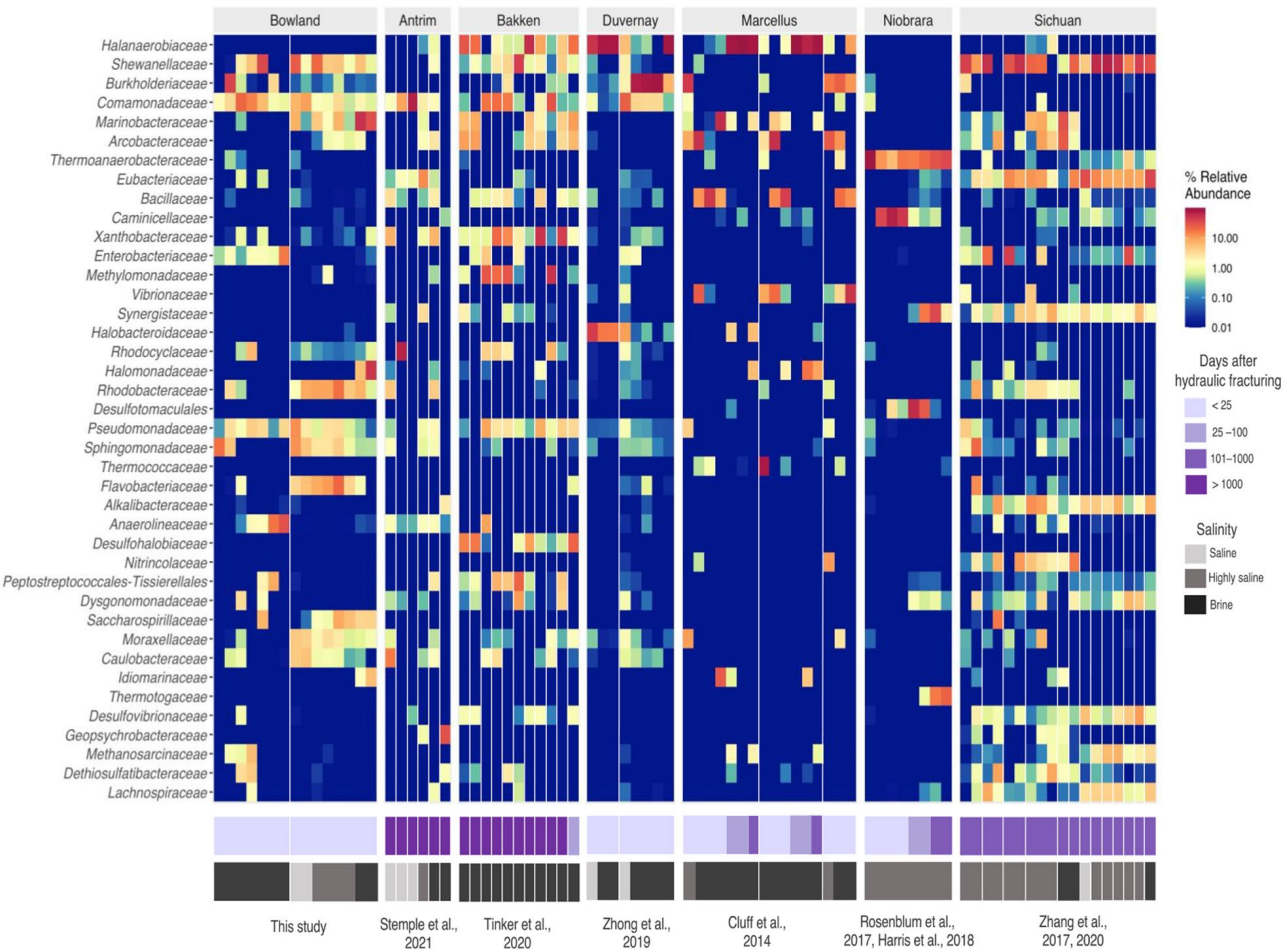
Heatmap displaying the top 30 most abundant families across all shale formations. Samples from different wells are indicated with separations within the facets. Annotations indicate the time of the sampling in a range of days after hydraulic fracturing and the salinity level following these categories saline (2000–10,000 mg/L), highly saline (10,000–60,000 mg/L) and brine (over 60,000 mg/L))

Members of the family *Halanaerobiaceae* were identified in 40% of the samples analyzed (Fig. 4), mostly in North American shale gas production fluids. *Halanaerobium* was the most persistent genus within this family; other genera included *Fuchsiella* and *Orenia*. *Halanaerobium* species have received attention as the dominant microbial taxon in flowback fluids recovered from numerous shale gas wells. *Halanaerobium* isolates have the metabolic potential to produce corrosive sulfide (via thiosulfate reduction), acids and form biofilms [61, 62]. Other studies have linked their presence with the potential for sulfide generation [42, 63] The family *Shewanellaceae* was detected in 55% of the samples, exhibiting greater abundance in the Bowland and Sichuan formations (Fig. 4). *Shewanella* species are also known sulfidogenic organisms via sulfur and thiosulfate reduction [64, 65]. Neither members of the *Halanaerobium* nor *Shewanella* genera were identified in the Niobrara formation (Fig. 4). Instead, sequences affiliated with *Thermoanaerobacter* were recovered in high abundance (Fig. 4). The genomes of bacteria affiliated with *Thermoanaerobacter* include rhodanese-encoding *asr* genes and have therefore been implicated in the reduction of thiosulfate to sulfide [49, 62]. Taken together, we note that the presence of putative sulfidogenic lineages is common in fractured shale production fluids regardless of salinity, suggesting the widespread potential for microbial souring in fractured shale gas.

**Fig. 5:**
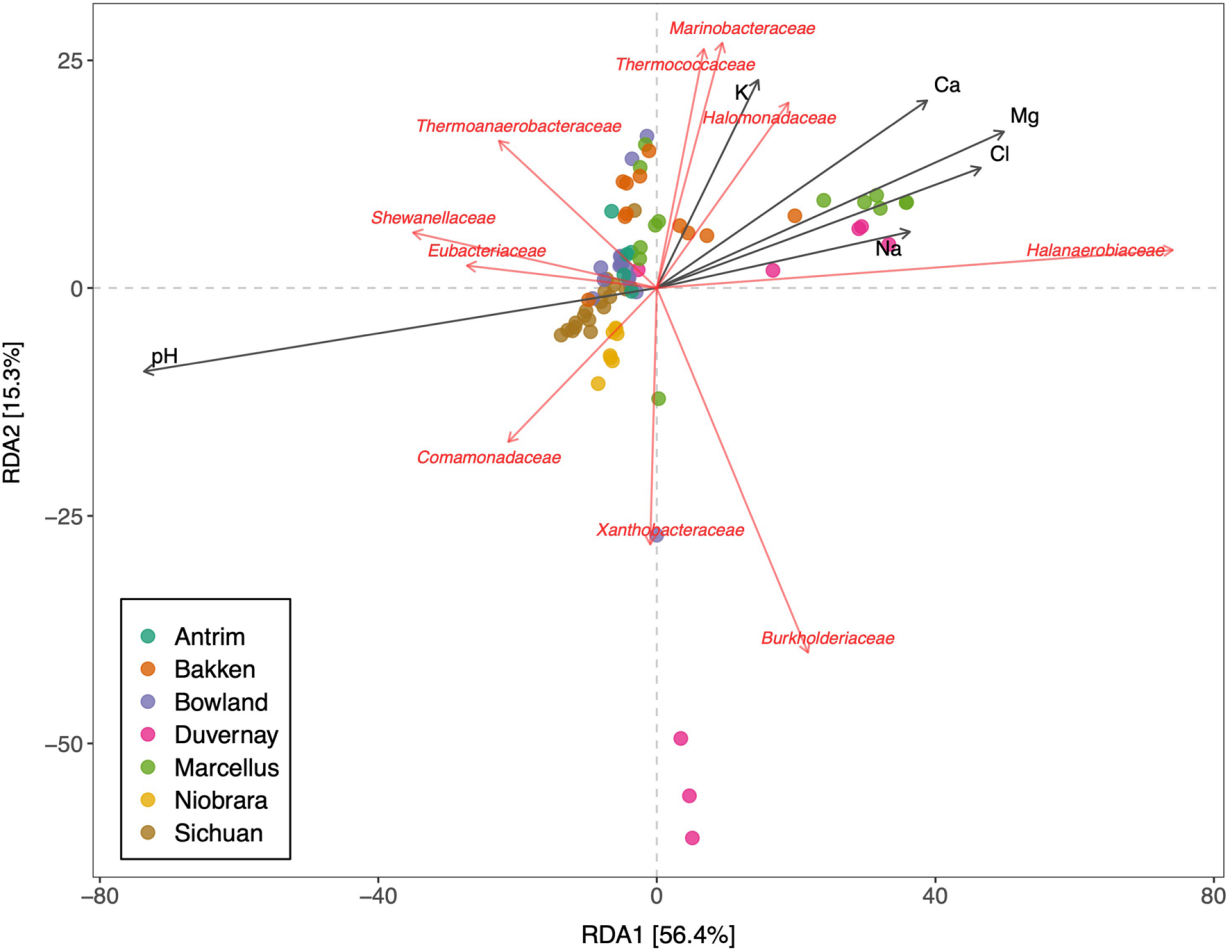
RDA biplot linking microbial composition and geochemistry in the fractured shale microbiome. Dots represent samples, black arrows environmental parameters and red arrows microbial families. The arrows indicate the direction of environmental variables associated with the different bacterial families

RDA analysis was applied to explore relationships between key environmental variables and microbial community structure across shales included in the meta-study. The first two axes of the RDA explained 71.7% of the variance (Fig. 5). Microbial communities from brine-level fluids such as the Marcellus, Duvernay and Bakken datasets were influenced by concentrations of Na, Cl, K and Ca. Thus, the abundance of halophilic families *Halanaerobiaceae* and *Halomonadaceae* was related to the presence of these ions. The microbial community from systems below brine level salinity, including Antrim, Bowland, Sichuan and Niobrara shales, did not exhibit clear relationships with the environmental factors included in the analysis. However, the distribution of members of the families *Shewanellaceae*, *Thermoanaerobacteraceae* and *Comamonadaceae* were characteristic of the lower salinity shales.

As noted above, the influence of salinity on community composition is strong, inducing physiological and thermodynamic constraints on the fractured shale microbiome [66]. We hypothesize that varying salinity levels lead to different organisms occupying similar niches in the shale microbiome, such as the potential to generate sulfide from thiosulfate or sulfur. As such, salinity levels potentially drive the microbial heterogeneity that was identified across shales. As an example, the distribution of members of the *Halanaerobiaceae* seems to be constricted to brine level systems (Additional file 1: Fig. S7A). In contrast, members of the family *Shewanellaceae* exhibit high abundance in saline or highly saline environments (Additional file 1: Fig. S7B). These findings are consistent with the dynamics in the lower-salinity STACK formation, where *Thermotogae* species were detected in high abundance [66]. Based on the findings of our 16S rRNA sequencing meta-study, lineages implicated with sulfide production are common regardless of salinity range, suggesting the sulfidogenic niche appears to be occupied throughout the formations considered here.

### A core fractured shale microbiome?

**Fig. 6:**
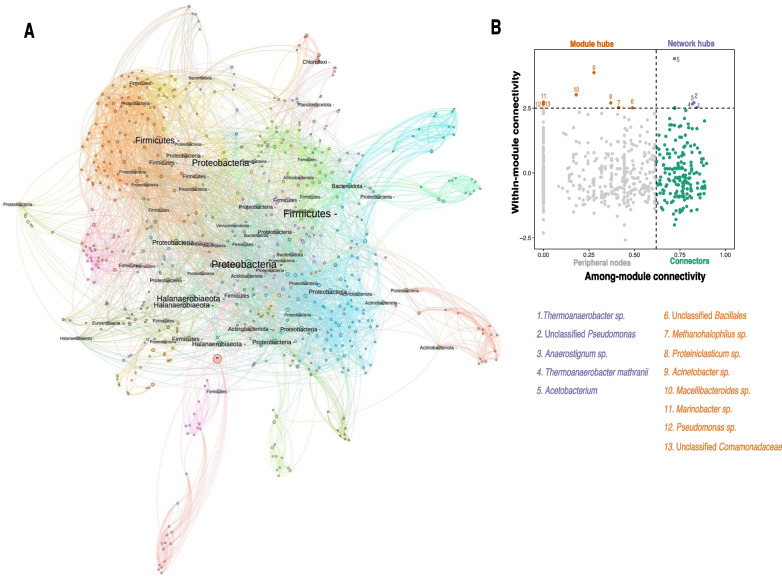
Network analysis of ASVs with abundance of ≥ 1% of the sequences in each given sample based on SPIEC-EASI. **A** Microbial co-occurrence network. Node’s size represents betweenness centrality. Nodes with betweenness centrality ≥ 1000 are annotated at phylum level. Distinct colors indicate distinct modules. Edges represent interactions between ASVs. **B** Classification of nodes based on among-module and within-module connectivity, network hubs (purple) and module hubs (orange) in the fractured shale microbiome

Identifying a “core microbiome” in the heterogeneous flowback and produced water samples derived from geographically distinct fractured shales is challenging. In an attempt to identify keystone taxa across these environments, a microbial network was constructed. The pruned dataset used for this analysis was comprised by 642 ASVs. The network attributes revealed a high modularity (0.753) with 24 modules, which had a low level of interactions among each other (clustering coefficient: 0.357). High levels of modularity have been associated with differences in locations and metabolic activity in aquifers contaminated with hydrocarbons [67]. We posit that this may also be the case in the fractured shale microbiome, where clusters might be related to differences in environmental variables, most notably salinity (Fig. 6A).

Nodes were classified based on their connectivity among and within modules as: peripherals, connectors, module hubs or network hubs (Fig. 6B). In ecological terms, peripherals can be considered as specialist, while connectors and module hubs are generalists and finally network hubs as super generalists [39]. As such, 8 nodes were identified as module hubs and 5 were network hubs (Fig. 6B). Network hubs have been proposed as putative keystone taxa, as their removal can cause a major change in the functioning and composition of the microbiomes [68]. Four of these network hubs correspond to ASVs affiliated to the genera *Thermoanaerobacter*, *Anaerostigum* and *Acetobacterium*. Members of these genera are acetogens, and can produce acetate, butyrate, or ethanol from H_2_ and CO_2_ [69–71]. The fifth network hub was affiliated with the genus *Pseudomonas*. Members of this genus have genes implicated in biofilm formation [72] and BTEX degradation [42]. The putative acetogenesis capacity present in most of the keystone taxa suggest that this pathway might have a key role sustaining the fractured shale microbiome. For instance, it is possible that the fermentation and subsequent complete oxidation of fracturing fluid additives stimulates acetogenesis by these keystone taxa, as labile organic carbon reserves become depleted. This hypothesis could be addressed through application of metagenomics in concert with other omics approaches.

### Perspectives on the fractured shale microbiome

Our study has shed light on the microbial ecology of fractured Bowland shale in the UK. In so doing, we contribute knowledge to the microbiology of these systems from outside of North America and China, where prior research has focused. In light of these new data, we highlight a broader range of microbial lineages that inhabit and seemingly proliferate in these engineered deep subsurface ecosystems. Notably, the lack of *Halanaerobium* spp. in the Bowland fractured shale systems, consistent with recent studies from fractured shales in the Sichuan and Niobrara formations [13, 15, 16], could have implications for the potential for souring and acid production during shale gas extraction of these formations. Where several studies have identified the capacity of strains of *Halanaerobium* to produce sulfide [62] and organic acids including under formation-relevant conditions [53], the potential for dominant lineages in fractured shales lacking dominant *Halanerobium* spp. remains unclear. Sequences affiliated to members of the family *Shewanellaceae* are dominant in fluids recovered from both the Bowland and Sichuan fractured shales, yet their metabolic potential in these environments is unknown. Similarly, sequences affiliated with the families *Thermoanaerobacteraceae* and *Caminicellaceae* are dominant in the Niobrara formation, and their metabolic activity in the fractured shale environment is not clear. *Shewanella* strains are known to produce sulfide [73] and form biofilms [74], thus they can be potentially deleterious for hydrocarbon production. In addition, *Thermoanaerobacter* strains are thiosulfate-reducers suspected to be involved in corrosion processes [75] and have been identified in biofilms.

We recognize our meta-study is necessarily limited to studies with both raw sequencing data and geochemical characterizations, and whilst the datasets were normalized prior to analysis, differences in sample processing, storage and sequencing technology can skew microbial compositional findings. However, datasets not included in this meta-analysis exhibited similar trends to those we highlighted here, most notably linking key microbial groups to salinity levels. For instance, production fluids derived from the hydraulic fractured Barnett shale have relative low TDS concentrations (8889 to 12,220 mg/L) and are dominated by the orders *Alteromonadales* (genus *Marinobacter*), while fluids with concentrations of TDS above detection limit are dominated by the class Firmicutes (genus *Halanaerobium*) [11]. *Halanaerobium* species were also prominent in fluids recovered from the Utica shale characterized by brine level salinities, with chloride concentration ranging from 40,522 to 95,100 mg/l [76].

Our work has highlighted the clear link between microbial community composition and salinity in fractured shale production fluids. However, a number of critical knowledge gaps remain. For instance, the degree to which the community composition of flowback fluids represents the in situ microbial community in fractured shale is unknown, and in particular the colonization of newly formed fractures by biofilm-forming microorganisms. In addition, most microbiological studies of shale gas production fluids to date have focused on community diversity, rather than activity. Few studies have interrogated the metabolic function of flowback fluid communities in relevant geochemical conditions (a notable exception is [46]. While diversity analysis (such as that employed here) can highlight key environmental drivers, it cannot identify active metabolic processes. The application of metagenomic sequencing to longitudinal flowback fluids has helped to constrain the metabolic potential of flowback fluid communities beyond inferences made from taxonomic assignment (e.g., [42, 76]), yet direct evidence for metabolic activity is lacking. Following on from this, the risk of corrosion and souring from microbial activity to shale gas extraction operations is unknown. A better understanding of these risks, and the factors influencing them, has the potential to enable shale gas extraction to be conducted more efficiently and with less environmental impact.

## Conclusions

This study investigated the biochemistry and microbiology of two hydraulically fractured exploration wells in the UK (Bowland shale). The geochemical and microbiological data were similar to those reported in other formations, in particular the Sichuan shale, and salinity was identified as a main geochemical trait influencing microbial diversity. Our findings indicate that Bowland shale flowback fluids have a moderate level of salinity and a high microbial diversity. Temporal shifts were identified in both wells, and changes in microbial community structure coincided with the increase in chloride concentrations. The effects of UV sterilisation were evident in the lack of detectable DNA recovered from the well it was used on, demonstrating the effectiveness of this approach in microbial control. The two wells showed distinct microbial communities, which we infer to be associated with UV sterilization, sampling conditions and salinity. However, taxa shared among the two wells revealed the persistence of bacteria with putative hydrocarbon metabolism and sulfidogenesis.

Our meta-analysis of the fractured shale microbiome sheds light on broader trends on the links between microorganisms and geochemistry of the produced fluids and highlights greater heterogeneity of these systems than previously observed.

Future efforts based on coordinated multi-omics approaches, as well as laboratory-based simulation studies, can begin to unpick the function and activity of the fractured shale microbiome, and in so doing uncover the role of microbial life in the deep terrestrial engineered subsurface. Understanding how microbial life interacts with these subsurface engineering activities may have significant implications for our reliance on the subsurface for transient storage of hydrogen and permanent storage of captured CO_2_ emissions, both of which are critical for achieving a sustainable, Net Zero future.

## Supplementary Information

**Additional file 1.** Supplermentary information.

**Additional file 2.** Complete meta-analysis dataset.

## Data Availability

Raw sequences were deposited in the NCBI under the bioproject PRJNA803344. Dataset used for meta-analysis including accession numbers corresponding to samples is detailed in Supplementary Data (Additional file 1). Code is available at https://github.com/NataliHB/ShaleMeta-analysis.git.
