# Supplementary material for: New microbiological insights from the Bowland shale highlight heterogeneity of the hydraulically fractured shale microbiome
Source: Environ Microbiome. 2023 Feb 28;18:14. doi: 10.1186/s40793-023-00465-1 (PMC9972762; doi:10.1186/s40793-023-00465-1)
Supplement: Supplementary file 1 — Additional file 1. Supplermentary information. [file 40793_2023_465_MOESM1_ESM.docx]

**SUPPLEMENTARY INFORMATION**

**New microbiological insights from the Bowland shale highlight heterogeneity of the hydraulically fractured shale microbiome**

Natali Hernandez-Becerra^1^, Lisa Cliffe^1,2^, Wei Xiu^1,3^, Christopher Boothman^1^, Jonathan R. Lloyd^1^ & Sophie L. Nixon*^1,2^

^1^Williamson Research Centre for Molecular Environmental Science, Department of Earth and Environmental Sciences, The University of Manchester, Manchester, UK.

^2^Manchester Institute of Biotechnology, The University of Manchester, Manchester, UK

^3^State Key Laboratory of Biogeology and Environmental Geology, China University of Geosciences (Beijing), Beijing, China

*Correspondence: sophie.nixon@manchester.ac.uk

**S 1**. Operational characteristics of the Bowland shale exploratory wells and sampling description.

| **Characteristic** | **Bowland-1** | **Bowland-2** |
| --- | --- | --- |
| Dates operational | Oct 2019 - Nov 2019 | Oct 2018 - Jan 2019 |
| Well name (operator ID) | PNR-2 | PNR-1z |
| Targeted formation | Upper Bowland Shale | Lower Bowland Shale |
| Formation composition | Siliciclastic system | Carbonate-dominated system |
| Well depth (m) | 2100 | 2300 |
| Number of fracturing stages | 5 | 42 |
| Biological control | UV disinfection | None |
| Surfactant | Sand | Sand |
| Acid | HCl | HCl |
| Viscosity modifier | Polyacrylamide | Polyacrylamide |
| Sampling point | Separator | Storage tanks |
| Number of samples | 7 | 8 |
| Sampling day since fracturing began | 60, 62, 65, 67,68, 82, 86 | 44, 45, 58, 65, 72, 80, 87, 94 |
| Storage on site | ~ hours at 4 °C | ~ weeks at 4 °C |
| Storage in labs | Immediately frozen -80 °C | Immediately frozen -80 °C |

**S 2**. Comparison of geochemical variables (ppm), alpha diversity metrics and DNA concentration (ug/ul) (mean ± standard error) in the two Bowland shale exploratory wells of this study. Statistically significant differences (student T test p < 0.05) between wells are indicated by superscript differing letter. BLD: below detection limit

|  | Bowland-1 | Bowland-2 |
| --- | --- | --- |
| Observed ASV | 76 (*± 22*) ^A^ | 245 (*± 48*) ^B^ |
| Shannon | 3.1 (*± 0.32*) | 3.5 (*± 0.34*) |
| DNA concentration | BDL | 0.0346 - 14.5 |
| pH | 6.2 (*± 0.04*) ^A^ | 7.1 (*± 0.15*) ^B^ |
| TOC | 9 *(± 1)* | 13 *(± 4)* |
| Chloride | 58 204 *(± 5 290)* ^A^ | 28 295 *(± 11 482)* ^B^ |
| Sodium | 13 792 *(± 1 018)* ^A^ | 8 894 *(± 3278)* ^B^ |
| Bromide | 479 *(± 50)* ^A^ | 174 *(± 64)* ^B^ |
| Potassium | 32 *(± 3)* | 23 *(± 7.5)* |
| Calcium | 1 955 *(± 216)* | 6 743 *(± 2 511)* |
| Magnesium | 886 *(± 96)* | 586 *(± 250)* |
| Iron | 24 *(± 4)* | 48 *(± 14)* |
| Strontium | 968 *(± 105)* | 567 (± 218) |
| Sulfate | 113 *(± 2)* | 101 (± 9) |

**S 3.** Non-metric multidimensional scaling (NMDS) plot produced for the flowback water samples with a stress value of 0.06. This plot was constructed using Bray-Curtis distance. Numbers denote days after hydraulic fracturing (A). Comparison of the group distances, permanova confirmed significant clustering by well (F.model= 2.44, Pr(>F)= 0.001).

**S 4.** Pearson correlations between the geochemical variables and the Bray Curtis distance matrix of the microbial community from the Bowland shale flowback samples.

| **Variable** | **Pearson correlation** | **p value** |  |
| --- | --- | --- | --- |
|  |  |  |  |
| **pH** | **0.325** | **0.005** |  |
| Na | 0.185 | 0.084 |  |
| **Cl** | **0.297** | **0.006** |  |
| **Br** | **0.372** | **0.001** |  |
| K | 0.210 | 0.067 |  |
| Ca | 0.029 | 0.395 |  |
| Mg | 0.213 | 0.077 |  |
| Fe | -0.089 | 0.648 |  |
| **Sr** | **0.233** | **0.031** |  |
| TOC | -0.322 | 0.982 |  |
| SO4 | -0.135 | 0.774 |  |

**S 5.** Major ions (ppm) and alpha diversity metrics of microbial communities in geographically distinct shale formations (mean ± standard error). Means followed by different superscripted letter are statistically different (one-way anova, Tukey test p < 0.05).

| **Formation** | **Observed ASVs** | **Shannon** | **pH** | **NaCl** | **Br** | **K** | **Ca** | **Mg** | **Fe** |
| --- | --- | --- | --- | --- | --- | --- | --- | --- | --- |
| Antrim | 77 ^A^  (*± 13*) | 3.3^A^  (*± 0.4*) | 7.3 ^A^  (± 0.2) | 40 536^CD^  (± 19911) | 219^A^  (± 106) | 644.2 ^A^  (± 355.3) | 3883^A^  (± 1658) | 354 ^AB^  (± 148) | 99^AB^  (± 35) |
| Bakken | 86 ^A^  (*± 9*) | 3.0^A^  (*± 0.1*) | 6.4 ^BC^  (± 0.1) | 257 100^A^  (± 16830) | 925^B^  (± 66) | 7289^B^  (± 861.3) | 20767^B^  (± 1883) | 1270 ^C^  (± 84) | 183^B^  (± 29) |
| Bowland | 102 ^A^  (*± 15*) | 3.2^A^  (*± 0.2*) | 6.7^ACD^  (± 0.1) | 53 432^CD^  (± 9319) | 272 ^A^  (± 71) | 403 ^A^  (± 153) | 4355 ^A^  (± 1389) | 385 ^AB^  (± 125) | 48 ^A^  (± 7) |
| Duvernay | 66 ^A^  (*± 23*) | 1.6 ^B^  (*± 0.4*) | 5.9^B^  (± 0.3) | 121 025^B^  (± 26583) | 488 ^A^  (± 114) | 1319 ^A^  (± 905.1) | 6822 ^A^  (± 2118) | 554 ^AB^  (± 128) | 71 ^A^  (± 23) |
| Marcellus | 16^B^  (*± 2*) | 1.8 ^B^  (*± 0.1*) | 6.2^BD^  (± 0.1) | 77 290^BD^  (± 8479) | 402^A^  (± 80) | 137.6 ^A^  (± 32.21) | 4468 ^A^  (± 1198) | 640 ^A^  (± 111) | 77^A^  (± 25) |
| Niobrara | 28^B^  (*± 5*) | 1.7 ^B^  (*± 0.2*) | 6.9^AC^  (± 0.1) | 15 265^C^  (± 727.8) | 143 ^A^  (± 10) | 220.1 ^A^  (± 45.38) | 556 ^A^  (± 125) | 70 ^B^  (± 15) | 28 ^A^  (± 8) |
| Sichuan | 66^A^  (± 5) | 2.7 ^A^  (± 0.1) | 7.3 ^A^  (± 0.1) | 25 901^C^  (± 2224) | 190 ^A^  (± 35) | 217.4 ^A^  (± 144) | 3234 ^A^  (± 1209) | 375 ^AB^  (± 117) | 37 ^A^  (± 8) |

**S 6.**  Relative abundance of dominant Phyla across geographically distinct shale formations. All Phyla that represent ≥ 5% of sequences from any sample are listed on the bar plot, the rest of phyla are grouped under “Other”.

**S 7.** Relative abundance of Halanaerobiaceae (A) and Shewanellaceae (B) across formations and their respective chloride concentration (ppm).
